# Integrative analysis of immune‐related signature profiles in eosinophilic chronic rhinosinusitis with nasal polyposis

**DOI:** 10.1002/2211-5463.13720

**Published:** 2023-11-07

**Authors:** Xuan Kan, Ruidi Guan, Jianwei Hao, Chunyuan Zhao, Yanan Sun

**Affiliations:** ^1^ Department of Otorhinolaryngology, Head and Neck Surgery The Second Affiliated Hospital, Harbin Medical University China

**Keywords:** CIBERSORT, eosinophilic chronic rhinosinusitis, nasal polyps, protein–protein interaction network, WGCNA

## Abstract

Eosinophilic chronic rhinosinusitis with nasal polyps (ECRSwNP) is a subtype of chronic rhinosinusitis (CRS) that is associated with the nasal cavity and sinus polyps, elevated levels of eosinophils, and dysregulated immune responses to environmental triggers. The underlying cause of ECRSwNP is not well understood, and few studies have focused on the unique features of this subtype of CRS. Our study integrated proteomic and transcriptomic data with multi‐omic bioinformatics analyses. We collected nasal polyps from three ECRSwNP patients and three control patients and identified 360 differentially expressed (DE) proteins, including 119 upregulated and 241 downregulated proteins. Functional analyses revealed several significant associations with ECRSwNP, including focal adhesion, hypertrophic cardiomyopathy, and extracellular matrix (ECM)–receptor interactions. Additionally, a protein–protein interaction (PPI) network revealed seven hub proteins that may play crucial roles in the development of ECRSwNP. We also compared the proteomic data with publicly available transcriptomic data and identified a total of 1077 DE genes. Pathways enriched by the DE genes involved angiogenesis, positive regulation of cell motility, and immune responses. Furthermore, we investigated immune cell infiltration and identified biomarkers associated with eosinophil and M2 macrophage infiltration using CIBERSORT and Weighted Gene Correlation Network Analysis (WGCNA). Our results provide a more complete picture of the immune‐related mechanisms underlying ECRSwNP, which could contribute to the development of more precise treatment strategies for this condition.

## Graphical abstract

Our multi‐omic study integrated the transcriptomic and proteomic data of eosinophilic chronic rhinosinusitis with nasal polyps (ECRSwNP), revealing eosinophil‐specific functional pathways and enabling the construction of protein–protein interaction networks. We also investigated immune cell infiltration and identified significant biomarkers. Our findings contribute to a better understanding of ECRSwNP, aiding the development of precise treatment strategies.

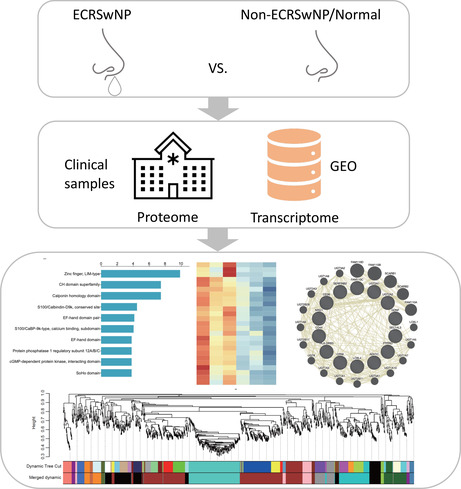

**Abbreviations**
CRS: chronic rhinosinusitis
DE: differentially expressed
ECM: extracellular matrix
ECRSwNP: eosinophilic chronic rhinosinusitis with nasal polyps
FC: fold‐change
FDR: false discovery rate
IL‐4/ IL‐5: interleukin 4/interleukin 5
MCC: maximal clique centrality
ME: module eigengene
MNC: maximum neighborhood component
PPI: Protein–protein interaction
WGCNA: Weighted Gene Correlation Network Analysis

Eosinophilic chronic rhinosinusitis with nasal polyps (ECRSwNP) is a type of chronic rhinosinusitis (CRS) that is characterized by the presence of polyps in the nasal cavity and sinuses, as well as elevated levels of eosinophils in sinus mucus and tissues. This distinct subtype of CRS is often associated with asthma, allergic rhinitis, and aspirin‐exacerbated respiratory disease [1, 2, 3]. Symptoms include nasal congestion, discharge, facial pain or pressure, and loss of sense of smell or taste. The polyps may cause obstruction of the nasal passages, leading to difficulty breathing through the nose, and patients may also experience recurrent sinus infections. The underlying cause of ECRSwNP is thought to involve a dysregulated immune response to environmental triggers (e.g., allergens or bacterial infections), which results in the recruitment of eosinophils and other inflammatory cells to the sinuses, leading to tissue damage, nasal polyp formation, and chronic inflammation [4]. However, the exact pathogenetic mechanisms underlying ECRSwNP are still not fully understood.

Compared with non‐ECRSwNP, ECRSwNP demonstrates a closer correlation with high objective disease severity, co‐morbid asthma, and the need for revision surgery. It is also characterized by Th2‐dominant inflammation, linked to allergies, and presents with severe nasal and sinus symptoms, which often requiring revision surgery, while non‐ECRSwNP exhibits Th1/Th17‐dominant inflammation, related to infections and autoimmune processes, resulting in milder symptoms and potentially better outcomes [5]. Immune cell infiltration is the characteristic manifestation of chronic inflammation, and previous studies have shown that nasal polyps from CRSwNP have higher amounts of immune cell infiltration. Eosinophils, a type of white blood cell involved in the immune response to parasites and allergens, are thought to play a key role in the development of ECRSwNP. In addition, various cytokines and chemokines have been implicated in the pathogenesis of ECRSwNP [6, 7]. Given the Th2‐dominant inflammation and the role of eosinophils in ECRSwNP, treatment strategies often target allergic and inflammatory pathways. This may involve the use of intranasal corticosteroids, systemic corticosteroids for acute exacerbations, and biologic therapies targeting specific Th2 cytokines like interleukin‐4 (IL‐4) or interleukin‐5 (IL‐5) [6]. Surgical intervention to remove polyps may also be necessary, but the recurrence rate tends to be higher, necessitating close follow‐up. Treatment for non‐ECRSwNP may be less reliant on allergy‐focused approaches and may involve a broader range of immunomodulatory therapies. Antibiotics or anti‐inflammatory agents targeting Th1 or Th17 pathways might be considered [8]. Thus, understanding the immune profiles and differentiation between ECRSwNP and non‐ECRSwNP is crucial for tailoring treatment approaches and managing patient expectations. It allows clinicians to choose appropriate medications and interventions, predict disease severity and recurrence risks, and provide more personalized care to individuals with CRS. Additionally, the presence or absence of eosinophils and specific cytokines can guide the use of emerging biologic therapies that target these pathways, further improving treatment outcomes.

Although several previous studies have characterized CRSwNP profiles, few studies have focused on the unique features of ECRSwNP. Thus, in this study, we characterized the mechanisms of ECRSwNP by integrating proteomic and transcriptomic data and completing bioinformatics analysis at a multi‐omic level (Fig. 1). We explored the key proteins, gene signatures, and pathways related to ECRSwNP. Specifically, we characterized the immune cell infiltration and identified the biomarkers that were significantly associated with eosinophil and M2 macrophage infiltration for ECRSwNP using CIBERSORT and Weighted Gene Correlation Network Analysis (WGCNA). Our results represent a more complete immune‐related picture of ECRSwNP and could eventually aid the development of precise treatment strategies.

**Fig. 1:**
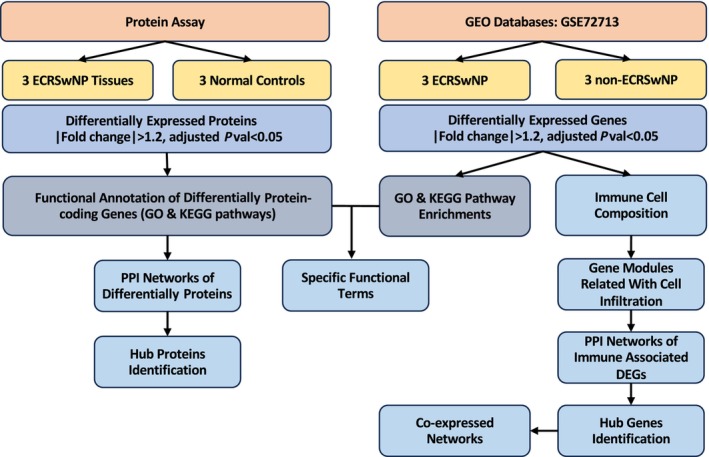
Overview of analysis pipeline.

## Materials and methods

### Patients and tissue collection

Samples were extracted from three cases of ECRSwNP tissue (as confirmed by clinical and pathological examination) and three cases of normal nasal mucosa tissue from beneath the inferior nasal concha. The pathological diagnosis criteria of ECRSwNP tissue were based on the proportion of inflammatory cells infiltrating the polyp tissue in immunohistochemistry. If the proportion of eosinophils in the tissue exceeded 10% of the total inflammatory cells, it was considered as the ECRSwNP tissue [9]. The samples were promptly frozen in liquid nitrogen for preservation after being collected from patients who had not received steroid treatment before their surgery. Experiments were performed following the ethical standards in the Declaration of Helsinki.

Patients were recruited from those who underwent sinusitis surgery and nasal septum deviation correction surgery at the Second Affiliated Hospital of Harbin Medical University. The studies involving human participants were reviewed and approved by the ethics committee of the Second Affiliated Hospital of Harbin Medical University (ID: YJSDW2022‐256). The patients/participants provided their written informed consent to participate in this study.

### Sample preparation and evaluation for protein assays

Samples were extracted using the Homogenate and SDT Lysis methods [10]. SDT buffer was added to each sample, and samples were then transferred to 2 mL tubes with an equal amount of quartz sand. Lysates were homogenized using a MP Fastprep‐24 Automated Homogenizer (6.0 M/S, 30s, two times). Homogenates were sonicated and then boiled for 15 min. After being centrifuged at 14 000 ***g*** for 40 min, supernatants were filtered with 0.22 μm filters. Each filtrate was quantified using the BCA Protein Assay Kit (P0012, Beyotime, Harbⅰn, China). Samples were stored at −20 °C. Twenty μg of proteins from each sample was mixed with 6X loading buffer and boiled for 5 min. The proteins were separated on 12.5% SDS/PAGE gel. Protein bands were visualized with Coomassie Blue R‐250 staining.

Two hundred μg of proteins for each sample was incorporated into 30 μL SDT buffer (4% SDS, 100 mm DTT, 150 mm Tris–HCl pH 8.0). The detergent, DTT, and other low‐molecular‐weight components were removed using UA buffer (8 m Urea, 150 mm Tris–HCl pH 8.5) using repeated ultrafiltration (Sartorius, Göttingen, Germany, 30 kD). Then, 100 μL iodoacetamide (100 mm IAA in UA buffer) was added to block reduced cysteine residues, and the samples were incubated in darkness for 30 min. The filters were washed with 100 μL UA buffer three times and then 100 μL 0.1 m TEAB buffer twice. Finally, the protein suspensions were digested with 4 μg trypsin (Promega, Madison, WI, USA) in 40 μL 0.1 m TEAB buffer overnight at 37 °C, and the resulting peptides were collected as filtrates. The peptide content was estimated using UV light spectral density at 280 nm with an extinction coefficient of 1.1 of 0.1% (g·L^−1^) solution that was calculated on the basis of the frequency of tryptophan and tyrosine in vertebrate proteins. One hundred μg peptide mixture of each sample was labeled using TMT reagent according to the manufacturer's instructions (Thermo Fisher Scientific, Waltham, MA, USA).

TMT‐labeled peptides were fractionated via RP chromatography using the Agilent 1260 infinity II HPLC. Peptide mixtures were diluted with buffer A (10 mm HCOONH4, 5% ACN, pH 10.0) and loaded onto an XBridge Peptide BEH C18 Column, 130 Å, 5 μm, 4.6  × 100 mm column. The peptides were eluted at a flow rate of 1 mL·min^−1^ with a gradient of 0–7% buffer B (10 mm HCOONH4, 85% ACN, pH 10.0) for 5 min, 7–40% buffer B during 5–40 min, 40–100% buffer B during 45–50 min, 100% buffer B during 50–65 min. The elution was monitored at 214 nm based on the UV light trace, and fractions were collected every 1 min for 5–50 min. The collected fractions were combined into 10 fractions and dried down via vacuum centrifugation at 45 °C.

### Mass spectrometry analysis

Each fraction was injected for nanoLC–MS/MS analysis. The peptide mixture was loaded onto the C18‐reversed phase analytical column (Thermo Fisher Scientific, Acclaim PepMap RSLC 50 μm × 15 cm, nano viper, P/N164943) in buffer A (0.1% Formic acid) and separated with a linear gradient of buffer B (80% acetonitrile and 0.1% Formic acid) at a flow rate of 300 nL·min^−1^. The linear gradient was determined as 6% buffer B for 3 min, 6–28% buffer B for 42 min, 28–38% buffer B for 5 min, 38–100% buffer B for 5 min, and hold in 100% buffer B for 5 min.

LC–MS/MS analysis was performed on a Q Exactive HF‐X mass spectrometer (Thermo Fisher Scientific) that was coupled to Easy nLC (Thermo Fisher Scientific) for 60 min. The mass spectrometer was operated in positive ion mode. MS data were acquired using a data‐dependent top10 method, dynamically choosing the most abundant precursor ions from the survey scan (350–1800 m/z) for HCD fragmentation. Survey scans were acquired at a resolution of 60 000 at m/z 200 with an AGC target of 3e6 and a maxIT of 50 ms. MS2 scans were acquired at a resolution of 15 000 for HCD spectra at m/z 200 with an AGC target of 2e5 and a maxIT of 45 ms, and the isolation width was 2 m/z. Only ions with a charge state between 2 and 6 and a minimum intensity of 2e3 were selected for fragmentation. Dynamic exclusion for selected ions was 30s. The normalized collision energy was 30 eV.

### Bioinformatics analysis for protein data

Raw MS/MS data files were processed using the MASCOT engine (Matrix Science, London, UK; version 2.6) embedded into Proteome Discoverer 2.2 and searched against the Uniprot HomoSapiens database, downloaded from http://www.uniprot.org. The search parameters included trypsin as the enzyme used to generate peptides with a maximum of two missed cleavages permitted. A precursor mass tolerance of 10 ppm was specified and 0.05 Da tolerance for MS2 fragments. Except for TMT labels, carbamidomethyl (C) was set as a fixed modification. Variable modifications were Oxidation (M) and Acetyl (Protein N‐term). A peptide and protein false discovery rate (FDR) of 1% was enforced using a reverse database search strategy [11]. Proteins with fold‐change (FC) > 1.2 and *P*‐value (Student's *t*‐test) < 0.05 were considered to be differentially expressed (DE) proteins.

At first, all protein sequences were aligned to the Uniprot_HomoSapiens_20367_20200226 database downloaded from NCBI (ncbi‐blast‐2.2.28 + −win32.exe), but only the sequences in top 10 and E‐value ≤ 1e‐3 were kept. Next, the GO term (database version: go_201504.obo) of the sequence with the top Bit‐Score by Blast2GO was selected. Then, the annotation from GO terms to proteins was completed using the Blast2GO Command Line. After the elementary annotations, InterProScan was used to search the EBI database by motif and then add the functional information of motif to the proteins to improve annotation. Further improvement of annotation and connection between GO terms was carried out using ANNEX. Fisher's Exact Tests were used to enrich GO terms by comparing the number of DE proteins and total proteins correlated with GO terms. Pathway analysis was performed using the KEGG database. Fisher's exact tests were used to identify the significantly enriched pathways by comparing the number of DE proteins and total proteins correlated with each pathway.

### RNA‐seq datasets

The transcriptome datasets for the ECRSwNP and NECRSwNP samples are publicly available in GEO repository GSE72713 [12]. The RNA‐seq datasets included nasal polyp tissue samples from three ECRSwNP and three non‐ECRSwNP patients. We downloaded six raw FASTQ files, including SRR2242952, SRR2242953, SRR2242954, SRR2242955, SRR2242956, and SRR2242957.

### RNA‐seq data processing and differential analysis

Raw sequenced reads from RNA‐seq datasets were quality‐tested using FASTQC (v0.11.8) (http://www.bioinformatics.babraham.ac.uk/projects/fastqc/) and mapped to the hg38 human genome using the STAR aligner (v2.5.3a) [13] with default parameters. Raw or TPM (Transcripts Per Kilobase Million) gene expression was quantified across all the exons of RefSeq genes with analyzeRepeats.pl in HOMER (v4.11.1) [14], which used the top‐expressed isoform as a proxy for gene expression. Differential gene expression was performed with the raw gene counts using the R package, DESeq2 (v1.24.0) [15], using replicates to compute within‐group dispersion. DE genes were defined as having a FDR < 0.05 and an absolute FC > 1.2 when comparing two experimental conditions. Principle component analyses were carried out on normalized gene counts using the R prcomp function. Heatmaps show *z*‐score normalized relative expression across conditions for each gene.

### Functional and pathway enrichment analyses

Over‐representation analysis was performed with the r package WebGestaltR [16] based on KEGG and GO (biological process, molecular function, and cellular component) pathways by setting FDR < 0.05 as the significance threshold, protein‐coding genes as the reference list, and a minimum number of genes in a category of five [16, 17]. The significantly enriched terms were ranked by the enrichment ratio scores for presentation in the bar plot.

### CIBERSORT analysis

To compare the immune cell composition of ECRSwNP and NECRSwNP samples, we used the CIBERSORT method to estimate cell fractions based on the gene expression profiles. This was implemented in the R immunedeconv::deconvolute (v2.1) [18]. We used the LM22 gene signature matrix, which includes 547 genes that can distinguish 22 types of human immune cells, as the annotation gene set. We then compared the relative proportions of immune cells between the two groups and calculated the correlations among various immune cells. The results were visualized using the Corrplot and ggplot2 r packages (https://github.com/taiyun/corrplot). The estimated scores for each cell type were compared between the two groups using Wilcoxon rank‐sum tests, and *P*‐values were calculated.

### Weighted gene co‐expression network analysis

We applied the r package WGCNA to explore the relationship between gene expression and immune cell infiltration [19]. We constructed an adjacency matrix based on the soft power value β and a similarity matrix calculated using Pearson correlation analysis among all genes. Subsequently, we transformed the adjacency matrix into a topological overlap matrix using a threshold soft power of five and generated a hierarchical clustering dendrogram to group genes with similar expression profiles into various modules (minModuleSize = 30, mergeCutHeight = 0.25). We calculated the module eigengene (ME) of each module and analyzed the correlations between ME and the relative proportion of different types of macrophages. The study focused on modules significantly correlated with eosinophil and M2 macrophage infiltration, which were considered hub modules. We then screened the genes from the hub modules by comparing them to the differential genes from RNA‐seq data and retained the overlap differential genes for further analysis.

The entire bioinformatics analysis was conducted using r software (v4.2.1).

### Protein–protein interaction analysis and identification of hub genes

We constructed protein–protein interaction (PPI) networks of DE proteins and immune cell‐associated DE genes using string (v11.5) [20]. A combined score of > 0.4 was used as the threshold for statistical significance. The PPI network was visualized using cytoscape (v3.9.1) [21] software, and highly interconnected gene modules were detected using the MCODE plug‐in for Cytoscape with a degree cutoff of two, node score cutoff of 0.2, K‐core of two, and max depth of 100. To identify hub genes in the PPI network, the cytoHubba Cytoscape plug‐in was used to score the genes based on their network features, using the results of maximum neighborhood component (MNC), maximal clique centrality (MCC), edge percolated component, degree, closeness, and radiality. Finally, a co‐expression network of hub genes and genes sharing common biological functions was built using GeneMANIA [22].

## Results

### Characterization of protein profiles of ECRSwNP

**Fig. 2:**
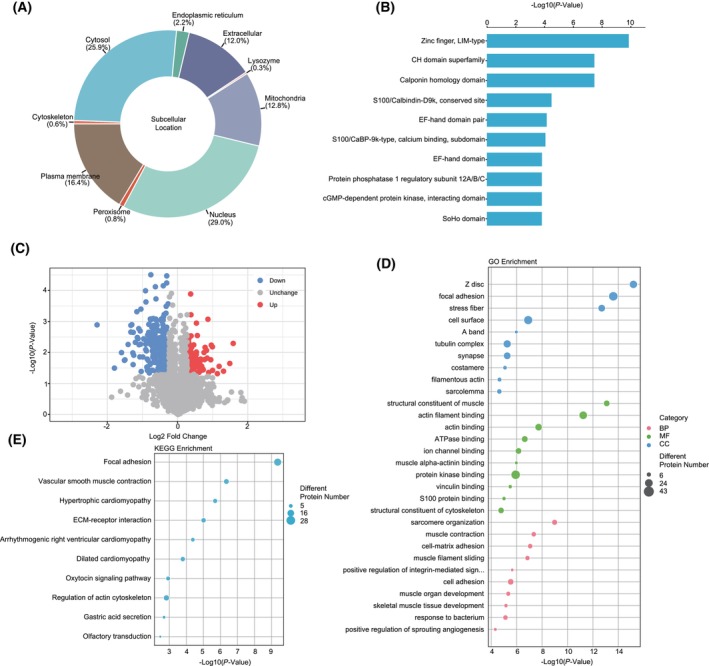
Dysregulated proteins in ECRSwNP and functional enrichment. (A) Protein subcellular localization prediction; (B) functional annotation of protein families, protein domains, and functional sites; (C) volcano plot of DE proteins; (D, E) GO and KEGG functional enrichment analysis.

We collected nasal polyps from three patients with ECRSwNP and three control patients and quantified the relative expression profiles of a total of 5560 proteins (Fig. 2A,B). By comparing the proteomic profiles of ECRSwNP samples to healthy controls, we identified a total of 360 DE proteins, including 119 upregulated and 241 downregulated proteins, in ECRSwNP. A volcano plot was used to visualize the changes in DE proteins (Fig. 2C), and details of the tested proteins are shown in Table S1.

### GO functional enrichment and KEGG pathway analyses of DE proteins in ECRSwNP

To investigate the mechanisms underlying ECRSwNP, we used DE proteins for GO functional enrichment and KEGG pathway analyses. Through GO enrichment analysis, we identified 102 upregulated terms that were significantly associated with ECRSwNP (FDR < 0.05), including 33 cellular components, 21 molecular functions, and 48 biological process terms. The top 10 most significant terms in each category are depicted in Fig. 2D, while the complete list of significantly over‐represented terms is provided in Table S2. Notably, the CC terms associated with the upregulated proteins in ECRSwNP were Z‐disc, focal adhesion, and stress fiber. The upregulated MF terms included structural constitute of muscle, actin filament binding, actin binding, and ATPase binding. The BP terms associated with the upregulated proteins were regulation of sarcomere organization, muscle contraction, cell‐matrix adhesion, and positive regulation of integrin‐mediated signaling pathway. In contrast, only one MF term, protein binding, was found to be associated with downregulated proteins in ECRSwNP.

We also performed KEGG pathway analysis to map the dysregulated protein‐coding genes to KEGG reference pathways and infer systemic biological behaviors. We found a total of 32 terms that were significantly over‐represented in upregulated proteins, which were mainly related to focal adhesion, vascular smooth muscle contraction, hypertrophic cardiomyopathy, and ECM–receptor interaction (Fig. 2E). The results of GO and KEGG pathway enrichment analyses are also demonstrated in Table S2.

### PPI network of upregulated proteins and hub proteins in ECRSwNP

**Fig. 3:**
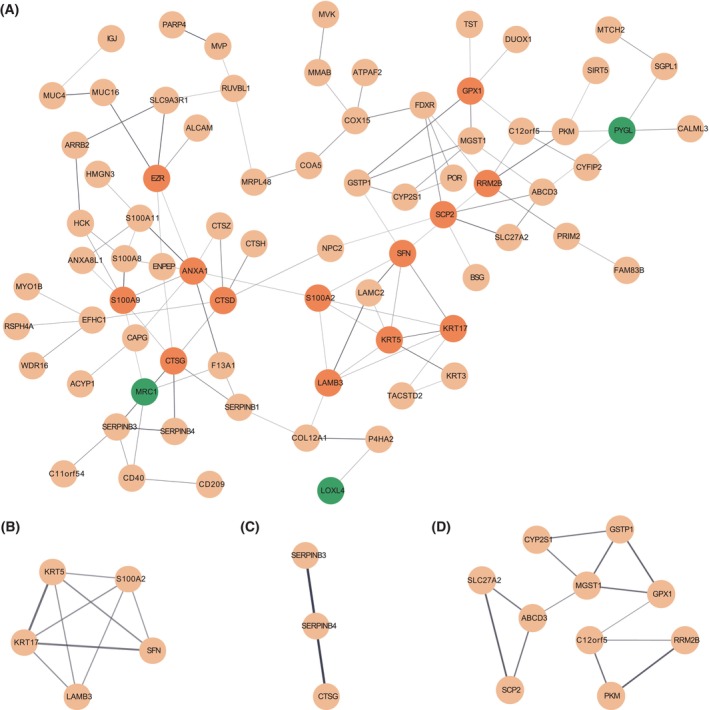
PPI network of upregulated proteins in ECRSwNP. (A) Main PPI networks; orange denotes the nodes with ≥ 5 degrees, and green denotes the nodes that overlap with DEGs from RNA‐seq; (B–D) subnetwork with highly connected nodes from (A).

To investigate the interactions among the DE proteins, we constructed a PPI network using Cytoscape. Specifically, we focused on the 119 upregulated proteins in ECRSwNP and filtered out isolated proteins to generate a PPI network comprising 77 nodes and 107 edges (Fig. 3A). Using the cytoHubba plug‐in in Cytoscape, we further identified three highly interconnected subnetworks consisting of proteins with similar biological functions (Fig. 3B–D). We also used the cytoHubba plug‐in to rank proteins in the network based on various parameters, such as their connection degrees, MCC, DMNC, and MNC, to screen for hub proteins that were densely associated with others. Subsequently, we identified seven hub proteins that were likely to play crucial roles in the development of ECRSwNP, including Annexin A1 (ANXA1), S100A2, 14‐3‐3 protein sigma (SFN), S100A9, Keratin 5 (KRT5), Keratin 17 (KRT17), and Laminin subunit beta‐3 (LAMB3).

### Characterization of gene profiles of ECRSwNP and non‐ECRSwNP

**Fig. 4:**
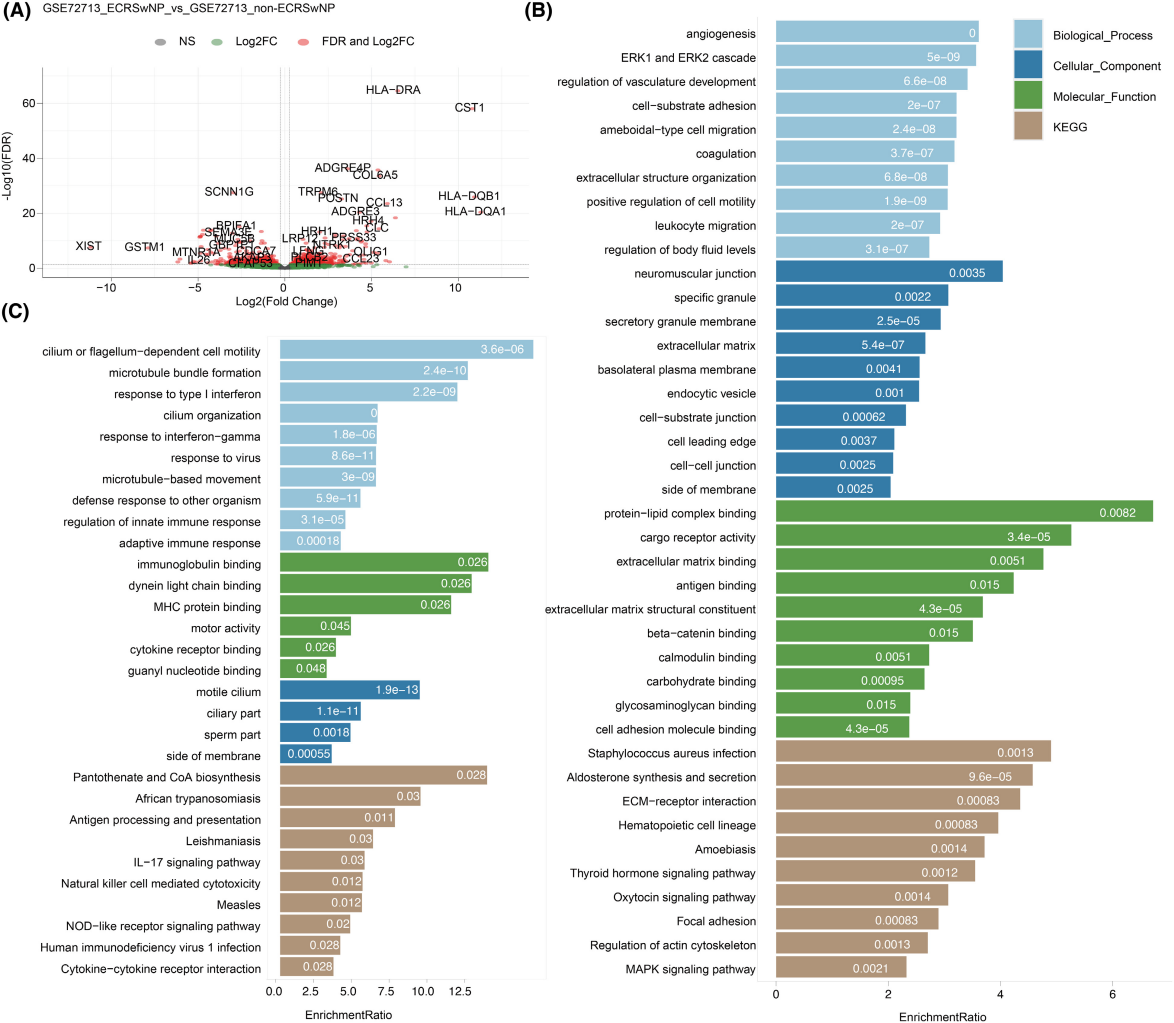
Dysregulated genes in ECRSwNP, based on RNA‐seq data and functional enrichment. (A) Volcano plot of DEGs; (B, C) enriched terms from GO and KEGG databases for up‐ and downregulated genes, respectively.

To gain further insight into the mechanisms underlying ECRSwNP, we integrated our proteomic data with publicly available transcriptomic data from GEO. Specifically, we obtained six raw sequencing datasets from a previously published study, GSE72713, comprising three samples from ECRSwNP patients and three samples from non‐ECRSwNP patients. By comparing the transcriptome profiles of nasal polyps between ECRSwNP and non‐ECRSwNP patients, we identified a total of 1077 DE genes, including 574 upregulated genes and 503 downregulated genes in ECRSwNP (Fig. 4A).

To assess the correlation between protein and gene expression levels, we compared the two sets of DE data and observed that the relative protein abundance of three genes—Lysyl Oxidase‐Like 4 (LOXL4), Mannose Receptor C‐Type 1 (MRC1), and Glycogen Phosphorylase L (PYGL)—was significantly upregulated in ECRSwNP. In addition, we found that the relative protein abundance of seven downregulated genes—GTPase, IMAP family member 1 (GIMAP1), Sodium Voltage‐Gated Channel Alpha Subunit 7 (SCN7A), Neurocalcin Delta (NCALD), Pleckstrin homology domain‐containing family F member 2 (PLEKHF2), Secreted Frizzled‐Related protein 4 (SFRP4), Cadherin 19 (CDH19), and Desmin (DES) was significantly reduced in ECRSwNP. Table S3 provides a comprehensive list of the differentially expressed genes (DEGs).

### GO functional enrichment and KEGG pathway analyses of DEGs from RNA‐seq data

To investigate the potential functions of the DEGs, we conducted GO functional enrichment and KEGG pathway analyses. Based on the enrichment ratio, we ranked the top 10 most enriched terms (Fig. 4B,C). The biological process (BP) terms associated with upregulated genes included: angiogenesis, regulation of vasculature development, ERK1 and ERK2 cascade, positive regulation of cell motility, ameboidal‐type cell migration, coagulation, extracellular structure organization, leukocyte migration, regulation of body fluid levels, and cell‐substrate adhesion. The upregulated genes were also significantly associated with many KEGG terms, including staphylococcus aureus infection, aldosterone synthesis and secretion, ECM–receptor interaction, hematopoietic cell lineage, amebiasis, thyroid hormone signaling pathway, oxytocin signaling pathway, focal adhesion, regulation of the actin cytoskeleton, and MAPK signaling pathway. In contrast, downregulated BP terms included cilium or flagellum‐dependent cell motility, microtubule bundle formation, response to type I interferon, cilium organization, response to interferon‐gamma, response to the virus, microtubule‐based movement, defense response to other organisms, regulation of innate immune response, and adaptive immune response. We also observed that the IL‐17 signaling pathway and cytokine–cytokine receptor interaction KEGG pathways were downregulated in ECRSwNP NP tissues.

We compared the functional terms enriched by DEGs and DE proteins and identified functional GO and KEGG terms that were specific to each group. The BP terms that were specific to DEGs were ERK1 and ERK2 cascade, leukocyte migration, and regulation of body fluid levels. The BP terms that were specifically enriched at the protein level included sarcomere organization and positive regulation of the integrin‐mediated signaling pathway. Some terms, such as angiogenesis and cell adhesion, were commonly found at both gene and protein levels. Interestingly, we identified several KEGG terms that were enriched at both gene and protein levels, including ECM–receptor interaction, focal adhesion, regulation of actin cytoskeleton, and oxytocin signaling pathway. Additionally, we found that the MAPK signaling pathway was specific to DEGs.

### Immune cell infiltration in ECRSwNP and non‐ECRSwNP samples

**Fig. 5:**
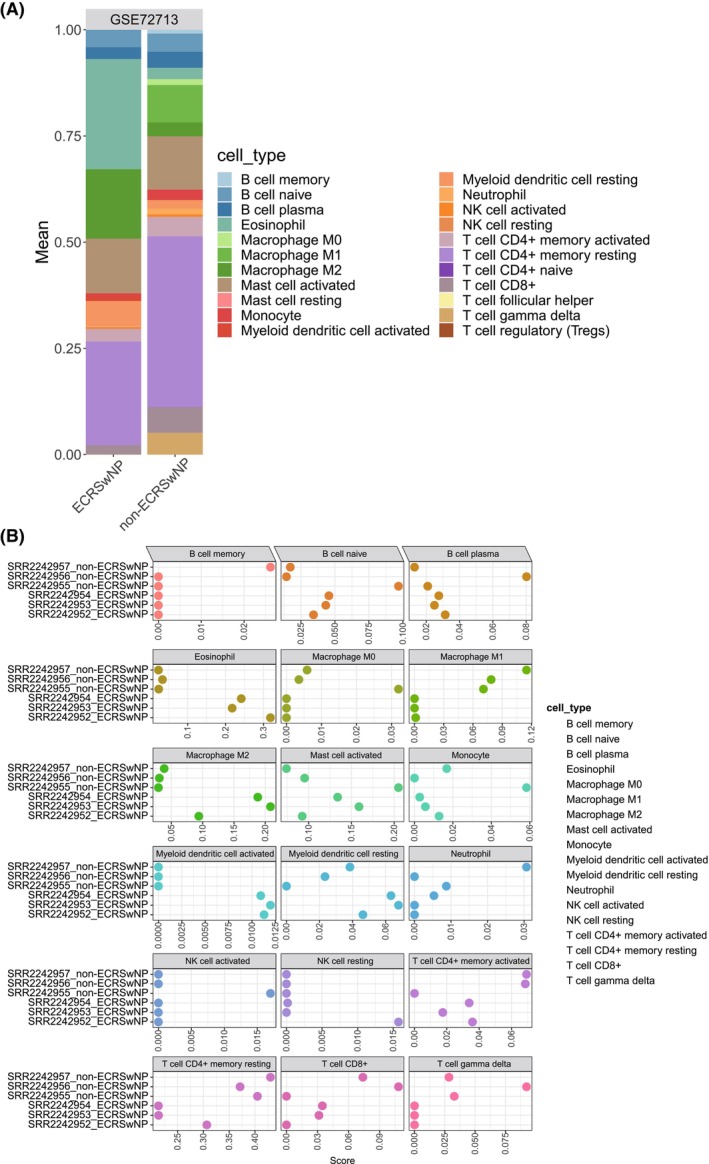
Immune infiltration landscape in ECRSwNP and non‐ECRSwNP samples. (A) Mean proportion of predicted immune cells in ECRSwNP and non‐ECRSwNP tissue; (B) predicted score of each sample for every cell type.

To assess the immune cell landscape in ECRSwNP and non‐ECRSwNP RNA‐seq samples, we used the CIBERSORT algorithm to estimate the relative abundance of 22 immune cell types (Fig. 5A). Subsequently, we compared the infiltration of immune cells between the two groups. ECRSwNP exhibited increased proportions of eosinophils (25.9% vs. 2.7%), M2 macrophages (16.3% vs. 3.3%), and activated myeloid dendritic cells (1.1% vs. 0%) in comparison with non‐ECRSwNP samples (Fig. 5B). Thus, these three immune cell types may play crucial roles in the development of ECRSwNP.

### Construction of co‐expression networks that are associated with immune cell infiltration

**Fig. 6:**
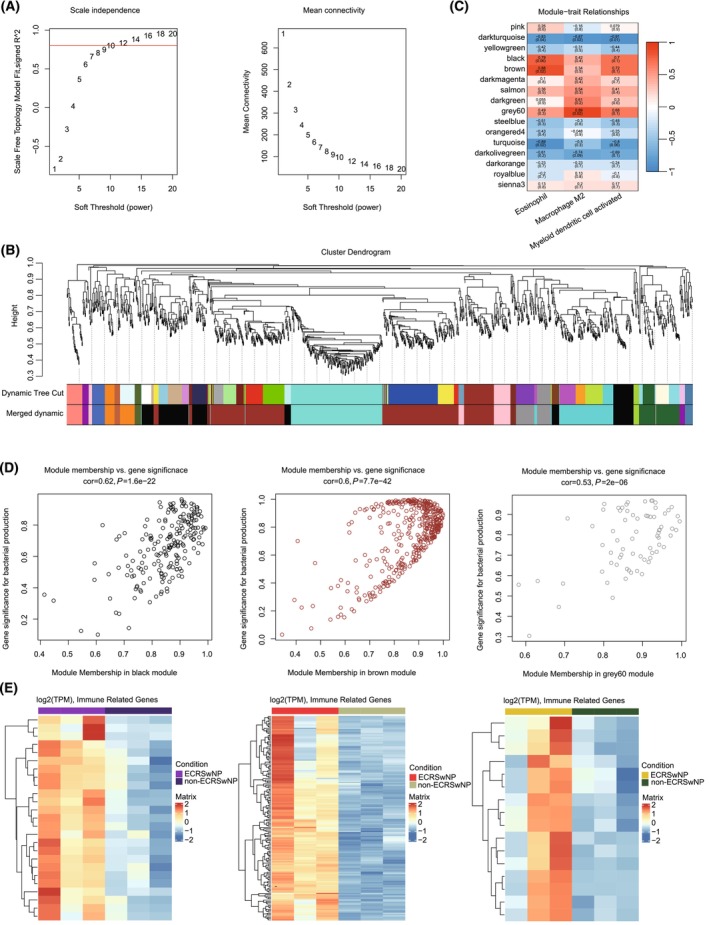
Construction of co‐expression network. (A) Selection of the soft threshold when the index of scale‐free topologies reaches 0.80 and analysis of the mean connectivity of 1–20 soft threshold power. (B) Establishment of co‐expressed gene modules based on hierarchical clustering algorithms. (C) Correlations between ME and infiltration of three cell types. The numbers inside each square indicate the correlation coefficients between infiltration and corresponding modules and the associated *P*‐values (red indicates a positive correlation, blue indicates a negative correlation, and the depth of the color indicates the degree of correlation). (D) Correlation of module membership in the black, brown, and grey60 modules; (E) heatmap of overlapping genes between module and RNA‐seq DGEs for the black, brown, and grey60 modules from right to left.

We used WGCNA to uncover the potential relationship between ECRSwNP and eosinophil and M2 macrophage infiltration. This allowed us to identify gene modules based on the connections between gene expression and to establish a scale‐independent topological network with a soft threshold power (β) of 12 and a scale‐free R2 of 0.8. The hierarchical clustering method was used to classify genes with analogous expression patterns into the same gene modules. A final 16 modules were obtained after merging the highly correlated ones (Fig. 6A–C).

To examine the association between the gene modules and cell infiltration, we used the ME as a representative of all genes within the module and computed the Pearson correlation coefficient between each module and eosinophil and M2 macrophage cell infiltration (Fig. 6D). We observed that the black (*R* = 0.79, *P*‐value = 0.06) and brown (*R* = 0.88, *P*‐value = 0.02) modules were significantly and positively correlated with eosinophil infiltration, while the grey60 module (*R* = 0.89, *P*‐value = 0.02) showed a significant positive correlation with M2 macrophage infiltration.

**Fig. 7:**
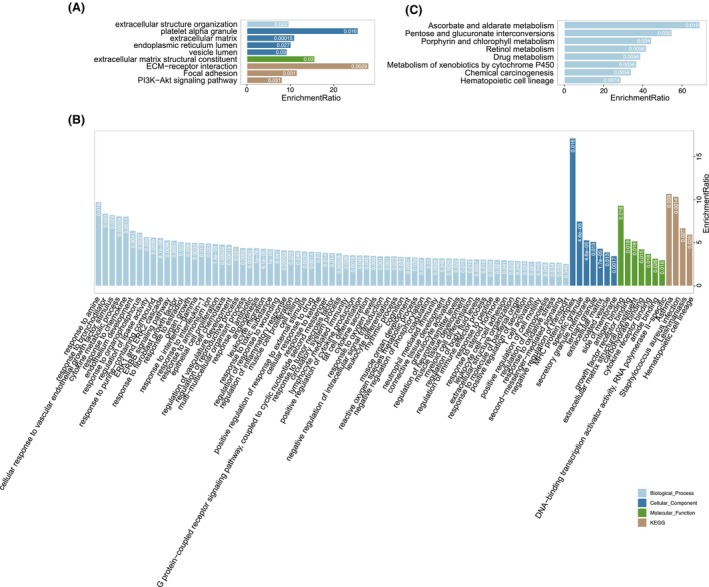
Functional enrichment of ECRSwNP‐associated differential genes from the black, brown, and grey60 modules (from A–C, respectively).

To investigate the functional significance of the three modules in ECRSwNP, we extracted the ECRSwNP‐associated module genes by comparing the gene members to the DEGs from RNA‐seq data. A total of 196 genes from the three modules were significantly upregulated in ECRSwNP, including 16 genes from the grey60 module, 25 genes from the black module, and 155 genes from the brown module (Fig. 6E). We then conducted a functional enrichment analysis in the GO and KEGG databases to annotate the functional relevance of the associated gene sets. Notably, the eosinophil infiltration‐associated module genes were enriched for different functional terms for the two modules. The black module was associated with extracellular structure organization, ECM–receptor interactions, focal adhesion, and the PI3K–Akt signaling pathway, while the brown module was associated with the ERK1 and ERK2 cascades, the ERBB signaling pathway, responses to chemokines, responses to interleukin−1, asthma, staphylococcus aureus infections, leishmaniasis, and hematopoietic cell lineages. The grey60 module associated with M2 macrophage was significantly enriched in KEGG pathways such as ascorbate and aldarate metabolism, pentose and glucuronate interconversions, and porphyrin and chlorophyll metabolism (Fig. 7).

### PPI networks of immune infiltration‐associated DEGs and identification of hub genes

**Fig. 8:**
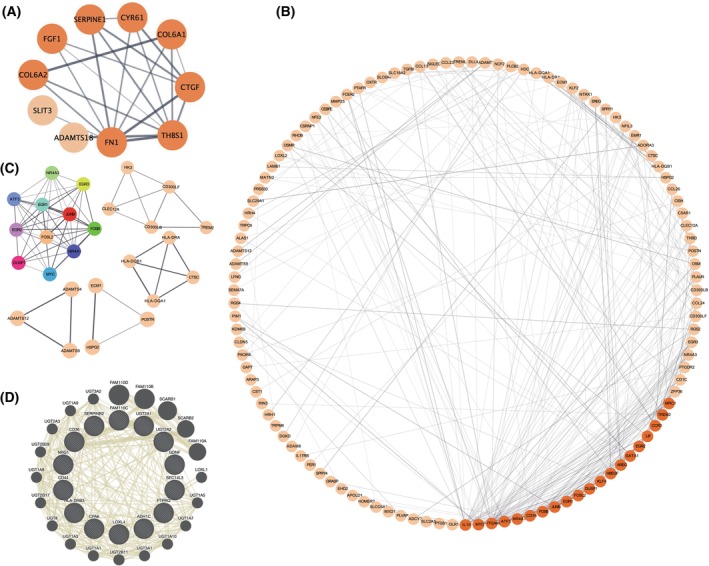
PPI networks of immune infiltration‐associated DEGs. (A, B) PPI networks in relation to the black and brown modules, respectively; (C) subnetworks identified from (B); (D) predicted PPI network for the M2 Macrophage‐related grey60 module.

In order to gain a better understanding of the regulatory relationships operating between module genes and to identify the hub genes that may have important immune functions in ECRSwNP, we constructed PPI networks for each module of interest using DEGs associated with immune cell infiltration in ECRSwNP. The DEGs that were upregulated in ECRSwNP based on RNA‐seq data were compared with module genes, and only the overlapping genes were retained for PPI construction. After removing isolated genes which had no interactions, we constructed two eosinophil‐associated PPI networks consisting of 151 nodes (10 for black and 141 for brown) and 319 edges (21 for black and 298 for brown; Fig. 8A,B). We then identified the top hub genes in each module that were densely connected with other genes in the network, based on degree and gene module membership (MM) index. For modules black and brown, we identified five top hub genes (FN1 and THBS1 for black; MYC, ATF3, and FOSB for brown). In addition, we identified five highly interconnected subnetworks from the eosinophil‐brown PPI network (Fig. 8C). These subnetworks consisted of genes with similar biological functions.

However, for the 16 macrophage M2‐associated genes from the grey60 module, we did not find highly correlated networks. To address this, we utilized GeneMANIA to explore the interaction of these genes by adding more association data, including protein and genetic interactions, pathways, co‐expression, co‐localization, and protein domain similarity. We identified a network containing 28 unique genes (16 input genes and 12 related genes) and 263 links, with 68.6% co‐expression and a 31.4% shared protein domain (Fig. 8D).

## Discussion

Eosinophilic chronic rhinosinusitis with nasal polyps is a chronic inflammatory disease affecting the nasal passages and sinuses. The pathogenesis of ECRSwNP is complex and involves the interplay of various genetic and environmental factors. Understanding the underlying mechanism could help identify new biomarkers, personalize treatment regimens, and improve prognoses. In this study, we characterized ECRSwNP in a complementary manner by integrating proteomic and transcriptomic data and systematically identifying and analyzing the proteins and genes associated with ECRSwNP.

By comparing the proteome profile of ECRSwNP NP tissues with healthy controls, we identified 360 dysregulated proteins and constructed PPI networks using proteins that were upregulated in ECRSwNP. From the highly connected networks, we identified seven hub proteins that may play important roles in the development of ECRSwNP, including ANXA1, S100A2, SFN, S100‐A9, KRT5, KRT17, and LAMB3. These proteins or their coding genes are all related to inflammatory and immune responses. For instance, ANXA1 is a calcium‐binding protein involved in the regulation of inflammation and immune responses [23]. ANXA1 has been implicated in the regulation of eosinophil infiltration and the production of inflammatory cytokines, which are key features of ECRSwNP. S100A2 is a calcium‐binding protein known to be involved in the regulation of cell differentiation and proliferation [24, 25, 26]. S100A9 is another member of the S100 family of calcium‐binding proteins that has been linked to the development of ECRSwNP [24]. It is involved in the regulation of inflammation and immune responses, and its upregulation has been observed in the nasal polyps of CRSwNP patients [27]. S100A9 has been shown to promote the activation of inflammatory cells and the production of cytokines and chemokines [26], which are key features of ECRSwNP. KRT5 and KRT17 are intermediate filament proteins that are expressed in epithelial cells [28]. KRT5 and KRT17 have been implicated in the regulation of cell proliferation and differentiation, and their upregulation may contribute to the abnormal growth of nasal polyps in ECRSwNP. LAMB3 is a component of laminin, a glycoprotein that is a major component of the extracellular matrix (ECM). Its upregulation may be associated with increased inflammation and tissue remodeling [29, 30]. LAMB3 is involved in the regulation of cell adhesion and migration, and its upregulation may contribute to the abnormal growth and invasion of nasal polyps in ECRSwNP.

Interestingly, there was only a minor overlap in the genes implicated in the transcriptome and proteome profiles. The three genes which did overlap (LOXL4, MRC1, and PYGL) are also related to inflammation. For instance, LOXL4 is a member of the lysyl oxidase family of enzymes, which are involved in the cross‐linking of ECM proteins such as collagen and elastin [31]. We found that LOXL4 was upregulated in the nasal polyps of ECRSwNP patients. Since its expression is positively correlated with the degree of tissue remodeling and fibrosis in the polyps, its upregulation in ECRSwNP may contribute to the chronic inflammation and tissue remodeling seen in the disease. MRC1 is a member of the C‐type lectin family of receptors that is expressed on the surface of macrophages and dendritic cells. MRC1 is involved in the regulation of immune responses, and its upregulation may contribute to the abnormal immune response and persistent inflammation in ECRSwNP [32].

Previous studies have shown that the immune cell infiltration of CRSwNP is higher than that in CRSsNP [33, 34]. In our study, we identified a similar immune microenvironment in ECRSwNP. Specifically, the rate of infiltration of eosinophils and M2 macrophages was higher in ECRSwNP when compared to non‐ECRSwNP. Eosinophils are a type of white blood cell that play a key role in the immune response to parasites and allergies [35]. In ECRSwNP, eosinophils are recruited to the nasal mucosa by chemokines and cytokines secreted by other immune cells, such as T cells and mast cells. Once in the tissue, eosinophils release a variety of pro‐inflammatory mediators, such as eosinophil‐derived neurotoxin, major basic protein, and eosinophil cationic protein, which can cause tissue damage and contribute to the development of nasal polyps. Eosinophils also secrete cytokines and chemokines that can recruit other immune cells to the site of inflammation, further amplifying the immune response in ECRSwNP [36]. M2 macrophages are another type of immune cell that have been implicated in the pathogenesis of ECRSwNP. M2 macrophages are a subset of macrophages that are involved in tissue repair and remodeling and are characterized by the production of anti‐inflammatory cytokines such as IL‐10 and TGF‐β [37]. In ECRSwNP, M2 macrophages are recruited to the nasal polyps by chemokines and cytokines secreted by other immune cells, such as eosinophils and Th2 cells. Once in the tissue, M2 macrophages produce pro‐fibrotic factors such as transforming growth factor‐beta and matrix metalloproteinases, which contribute to tissue remodeling and fibrosis in the nasal polyps. The accumulation of eosinophils and M2 macrophages in the nasal polyps of ECRSwNP patients suggests that these immune cells play an important role in the pathogenesis of the disease. The pro‐inflammatory mediators and cytokines released by eosinophils can cause tissue damage and contribute to the development of nasal polyps, while the pro‐fibrotic factors produced by M2 macrophages can lead to tissue remodeling and fibrosis. Targeting these immune cells and their associated cytokines and chemokines may therefore represent a promising approach for the treatment of ECRSwNP.

After constructing PPI networks, we found that the second and first modules were highly associated with eosinophils and M2 macrophages, respectively. We found that most of the significantly enriched GO terms and KEGG pathways were related to inflammatory and immune responses. For example, ECM organization, ECM–receptor interaction, focal adhesion, and several signaling pathways, including PI3K‐Akt, ERK1/2, and ERBB, were all implicated in the pathogenesis of ECRSwNP. ECM organization and ECM–receptor interactions are critical for cell adhesion, migration, and differentiation. Alterations in the ECM structure and its receptors were observed in ECRSwNP, which may contribute to the formation and maintenance of nasal polyps. Focal adhesion is also involved in cell adhesion and migration, and its dysregulation may contribute to the excessive accumulation of inflammatory cells and tissue remodeling in ECRSwNP. The PI3K‐Akt and ERK1/2 cascade signaling pathways are involved in cell survival, proliferation, and differentiation. The dysregulation of these pathways could potentially increase cell survival and proliferation, which may also contribute to the formation and maintenance of nasal polyps [38, 39]. The ERBB signaling pathway is involved in cell proliferation and differentiation, and its dysregulation may contribute to the proliferation and survival of inflammatory cells and tissue remodeling in the disease. Several metabolic pathways, including ascorbate and aldarate metabolism, pentose and glucuronate interconversions, and porphyrin and chlorophyll metabolism, were also found to be related to ECRSwNP. These pathways are involved in the synthesis and degradation of important cellular components, and their dysregulation may also contribute to the development of nasal polyps.

Some limitations in our study should be acknowledged. First, the proteome and transcriptome data acquired from tissue from different patients. The observed changes may potentially be confounded by individual differences. Second, as our clinical samples for proteomic analysis were matched ECRSwNP and normal tissue, they were not specially targeted on eosinophil characters. Although we have discovered the eosinophil‐related functions by comparing to transcriptome results, a protein‐level comparison in the future study between eosinophilic and non‐eosinophilic nasal polyps could potentially yield more profound insights into eosinophil‐related characteristics.

Third, the sample size of our study was relatively small. This may limit statistical power for uncovering differences between ECRSwNP tissue and the control tissue. Thus, further research in a larger cohort is needed to confirm our results and fully elucidate the immune mechanisms underlying ECRSwNP.

In summary, the pathogenesis of ECRSwNP is complex and involves several molecular pathways and metabolic processes. Our study presents a comprehensive immune‐related picture of ECRSwNP and could eventually help aid in the development of precise treatment strategies.

## Conflict of interest

The authors declare no conflict of interest.

### Peer review

The peer review history for this article is available at https://www.webofscience.com/api/gateway/wos/peer‐review/10.1002/2211‐5463.13720.

## Author contributions

XK wrote the manuscript. RG and JH did the main experiment. XK and YS designed the whole experiment. CZ helped to revise the article. All authors read and approved the final manuscript.

## Funding information

The author(s) received no financial support for the research, authorship, and/or publication of this article.

## Supporting information


**Table S1.** Protein quantification and differential analysis list.

**Table S2.** GO and KEGG enriched terms from differential expressed proteins.

**Table S3.** Gene quantification and differential analysis list.

## Data Availability

The RNA‐seq datasets used in this study can be found in GEO (GSE72713). The protein datasets used and analyzed in this study can be obtained from the corresponding author upon reasonable request.
